# Physicochemical Changes and Resistant-Starch Content of Extruded Cornstarch with and without Storage at Refrigerator Temperatures

**DOI:** 10.3390/molecules21081064

**Published:** 2016-08-15

**Authors:** David Neder-Suárez, Carlos A. Amaya-Guerra, Armando Quintero-Ramos, Esther Pérez-Carrillo, María G. de J. Alanís-Guzmán, Juan G. Báez-González, Carlos L. García-Díaz, María A. Núñez-González, Daniel Lardizábal-Gutiérrez, Jorge A. Jiménez-Castro

**Affiliations:** 1 Departamento de Investigación y Posgrado, Facultad de Ciencias Biológicas, Universidad Autónoma de Nuevo León, Ciudad Universitaria, San Nicolás de los Garza 66450, Mexico; neder_david@yahoo.com.mx (D.N.-S.); numisamaya@hotmail.com (C.A.A.-G.); maria.alanisgm@uanl.edu.mx (M.G.d.J.A.-G.); juan.baezgn@uanl.edu.mx (J.G.B.-G.); clgarciadiaz@gmail.com (C.L.G.-D.); maria.nunezgn@uanl.edu.mx (M.A.N.-G.); 2 Departamento de Investigación y Posgrado, Facultad de Ciencias Químicas, Universidad Autónoma de Chihuahua, Circuito Universitario s/n Campus Universitario 2, Chihuahua 31125, Mexico; jajimenez@uach.mx; 3 Centro de Biotecnología-FEMSA, Escuela de Ingenería y Ciencias, Tecnológico de Monterrey, Av. Eugenio Garza Sada 2501 Sur, Monterrey 64849, Mexico; perez.carrillo@itesm.mx; 4 Centro de Investigación en Materiales Avanzados, S. C. Avenida Miguel de Cervantes 120, Complejo Industrial Chihuahua, Chihuahua 31109, Mexico; daniel.lardizabal@cimav.edu.mx

**Keywords:** extrusion cooking, resistant starch, thermal properties, viscoelastic properties, cornstarch

## Abstract

Effects of extrusion cooking and low-temperature storage on the physicochemical changes and resistant starch (RS) content in cornstarch were evaluated. The cornstarch was conditioned at 20%–40% moisture contents and extruded in the range 90–130 °C and at screw speeds in the range 200–360 rpm. The extrudates were stored at 4 °C for 120 h and then at room temperature. The water absorption, solubility index, RS content, viscoelastic, thermal, and microstructural properties of the extrudates were evaluated before and after storage. The extrusion temperature and moisture content significantly affected the physicochemical properties of the extrudates before and after storage. The RS content increased with increasing moisture content and extrusion temperature, and the viscoelastic and thermal properties showed related behaviors. Microscopic analysis showed that extrusion cooking damaged the native starch structure, producing gelatinization and retrogradation and forming RS. The starch containing 35% moisture and extruded at 120 °C and 320 rpm produced the most RS (1.13 g/100 g) after to storage at low temperature. Although the RS formation was low, the results suggest that extrusion cooking could be advantageous for RS production and application in the food industry since it is a pollution less, continuous process requiring only a short residence time.

## 1. Introduction

Starch is a complex carbohydrate composed of glucose units consisting of amylose and amylopectin [1,2] and is widely used in food production. However, current market trends demand functional ingredients with health benefits, and starch transformed into resistant starch (RS) shows advantages in a diverse range of applications. RS has modified starch or starch fractions that are indigestible in the small intestine, so it is similar to dietary fiber and shows similar health benefits [3,4]. RS is classified as physically inaccessible starch (type 1), raw starch granules and high-amylose starch (type 2), retrograded gelatinized starch or dispersion granules (type 3), and chemically modified starch (type 4) [4,5,6,7].

RS yields vary according to the hydrolysis process used; e.g., HCl (12%–25%) [8,9], lactic acid (2%–8%) [10], citrate (41%), and phosphate (25%–70%) [11,12]. Alternative hydrothermal processes performed at 110 °C over three days yielded 12% RS [13] and up to 25% in an acidic environment [14,15,16,17]. However, such processes are lengthy, show high energy cost, and produce pollutants [18] and in some cases even require batch type operations that produce low yields [13,18]; therefore, such processes are impractical for industrial scaling.

Extrusion cooking has conventionally been used to obtain a variety of extruded products because it is a continuous process, it is versatile, and it shows short processing times, high productivity, and energy efficiency [4,5,18,19,20,21]. The extrusion cooking process (ECP) has been used to produce RS [5,13,20,22,23,24] at low yields under certain extrusion conditions [20]. Under some conditions, however, the ECP did not produce any RS [13] due to the effect of extrusion variables such as extrusion temperature, moisture content, and screw speed during processing [13,20,22,25]. Certain combinations of these parameters result in starch retrogradation or excessive dextrinization [5,7,9,26,27,28,29,30,31], which limit the formation of RS. Cornstarch has previously been extruded at high screw speeds without RS detection [13]. High-moisture-content banana and oat starches have generated low-yield RS due to a low gelatinization and component release, respectively [20,24]. Wheat flour subjected to high-temperature extrusion showed low levels of RS due to high dextrinization during the ECP [5], rendering the starch unusable for RS generation [23,32]. Other studies [22,23] have reported the production of more than 10% RS from mango and banana starches at different moisture contents and extrusion temperatures, suggesting that more studies are needed to evaluate the influence of extrusion variables in other ranges of the process on RS formation. Optimizing the moisture content, extrusion temperature, and screw speed is critical for the formation of RS. Therefore, the aims of this study were to evaluate and optimize the ECP conditions and to determine their effects on the physical and chemical changes in RS formed from cornstarch.

## 2. Results and Discussion

### 2.1. Chemical Composition of Cornstarch

The proximate composition of the native cornstarch is shown in Table 1. The moisture, ash, protein, and fat contents were 10.9%, 0.03%, 0.1%, and 0.05%, respectively. Similar results have previously been reported [23,33]. The RS content was 0.670 g/100 g, similar to the finding reported by Shi and Gao [34].

### 2.2. Model Fitting

The influences of the extrusion temperature, feed moisture content, and screw speed on the physical and chemical properties of cornstarch are shown in Table 2 and Table 3, respectively. The analysis of variance for all the responses indicated an adequate adjustment without significant lack of fit, except for the RS content and the WSI of the extruded starch before storage (EBS) (Table 4). The regression coefficients were obtained by fitting the experimental data to the second-order model (Table 5), and the models gave satisfactory R^2^ values.

### 2.3. Water Absorption Index (WAI)

**Figure 1:**
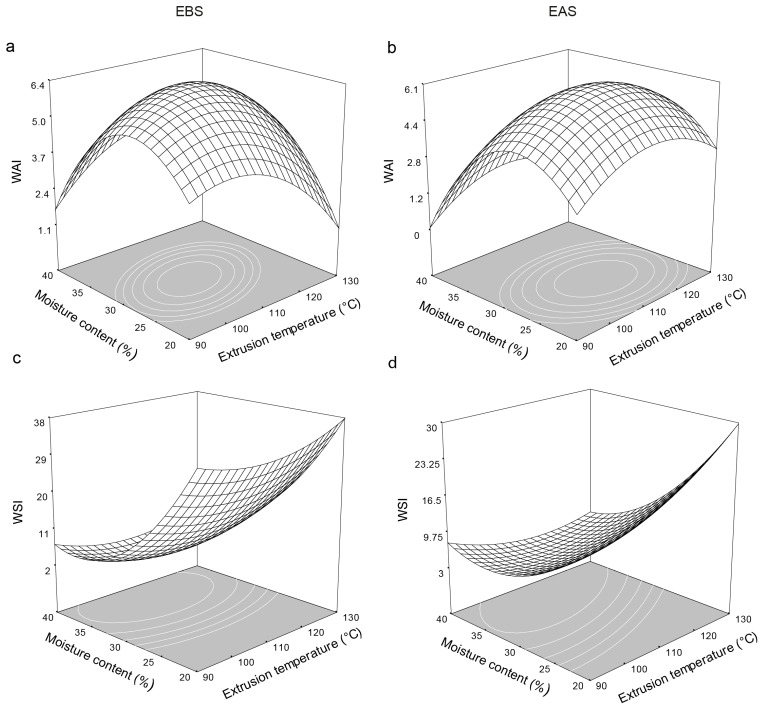
Effect of extrusion temperature and moisture content on water absorption index (WAI) and water solubility index (WSI) at a screw speed of 280 rpm on extruded flours before the storage (EBS) and extruded flours after the storage at 4 °C for 120 h (EAS). (**a**)—WAI of EBS; (**b**)—WAI of EAS; (**c**)—WSI of EBS and **d**—WSI of EAS.

The WAI is an indicator of the capacity of flour to absorb water, which depends on the availability of hydrophilic groups that bind to water molecules and on the capacity of macromolecules to form a gel [35]. The values ranged from 2.7 to 6.8, higher than that of the native starch (1.8). The screw speed and moisture content significantly affected the WAI of the EBS, while the extrusion temperature and moisture content significantly affected at the WAI of the extruded starch after storage (EAS) (Table 4). Figure 1a,b shows the effects of the extrusion temperature and the moisture content on the WAI of the EBS and EAS extrudates, respectively. The WAI of the EBS extruded at 106 °C and a screw speed of 249.5 rpm increased to a maximum of 6.33 at a moisture content of 28.5% with increasing extrusion temperature and moisture content (Figure 1a). The EAS showed a similar trend (Figure 1b) with marked effects of moisture content and extrusion temperature (Table 4). The adjusted model shows that the maximum WAI for the EAS containing 27.3% moisture and extruded at 112 °C and 267.3 rpm was similar to the WAI for the EBS. The changes in the WAI of the EBS and EAS are attributed mainly to the extrusion temperature and the high moisture content, which both favor mixing in the extruder thereby increasing starch granule swelling and starch gelatinization, which increase the capacity of the starch to absorb water and increase the starch viscosity [36]. Figure 1a shows at low moisture content the WAI was influenced by temperature, at low temperatures, starch gelatinization was low minimizing the amount of damage to the starch structure [24,37]. While at high extrusion temperatures cause crystal melting of amylopectin molecules, generating more starch damage (dextrinization and destruction of starch structure), decreasing the WAI [23,25].

### 2.4. Water Solubility Index (WSI)

The WSI is a parameter for measuring the degradation of starch. Lower WSI values indicate that less starch is degraded and that the extruded products contain fewer soluble molecules [38]. Figure 1c shows that the WSI of the EBS containing 40.4% moisture and extruded at 110.3 °C and 348 rpm decreased with increasing moisture content to a minimum of 2.13. The WSI of the EAS shows a similar trend (Figure 1b). The adjusted model shows a saddle point for the EAS containing 70.97% moisture and extruded at 204.67 °C and 283.47 rpm, which explains 85% of the variability in the data. The WSI decreased at high moisture contents due to partial starch gelatinization during extrusion, which prevents the release of components [20,22,39]. High moisture contents in feed into the extruder possibly act like lubricants during transportation of solid through the barrel, minimizing the phenomenon of starch gelatinization and thus the associated changes in the rheological and functional properties (WSI). Similar results have been reported by Bhattacharya et al. [40]. Treatments under low-moisture conditions may restrict the flow of material into the extruder barrel thereby incrementing the amount of friction and the viscosity of the starch [36], which increases the number of degraded starch granules formed at high temperatures thereby forming significantly more water-soluble products [41]. This phenomenon is caused by greater fragmentation of the starch, a process known as dextrinization, during extrusion thereby increasing the percentage of gelatinized starch [24,25,35,37].

### 2.5. Viscoelastic Properties

**Figure 2:**
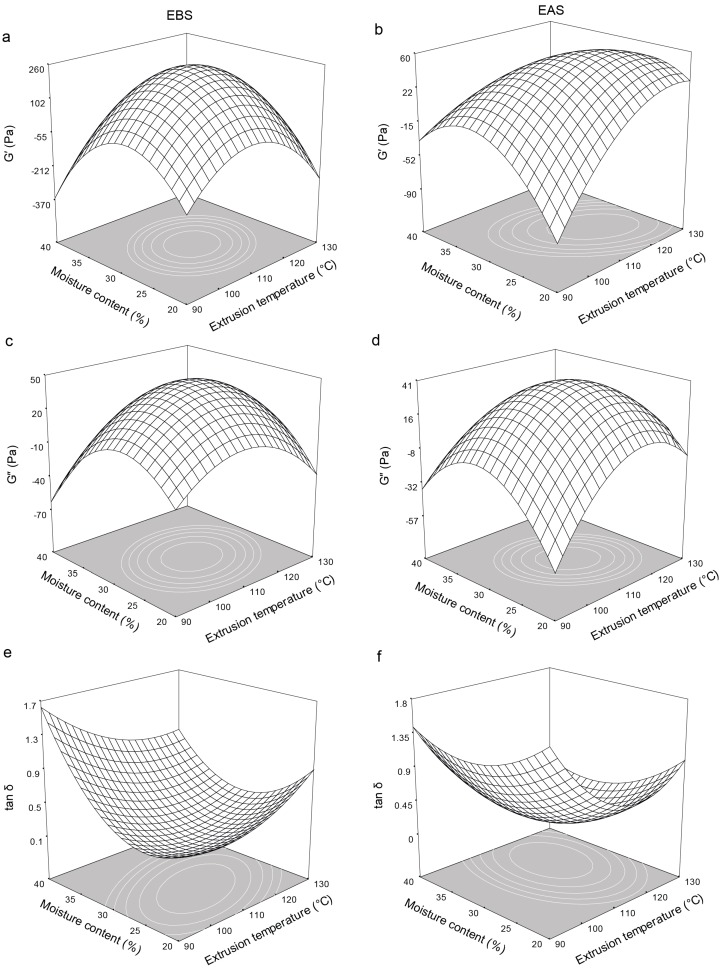
Effect of extrusion temperature and moisture content on: loss modulus (G’), stored modulus (G”) and the ratio of loss modulus/storage (tan δ) at a screw speed of 280 rpm on extruded flours before the storage (EBS) and extruded flours after the storage at 4 °C for 120 h (EAS). (**a**)—G’ of EBS; (**b**)—G’ of EAS; (**c**)—G” of EBS; (**d**)—G” of EAS; (**e**)—tan δ of EBS; (**f**)—tan δ of EAS.

**Figure 3:**
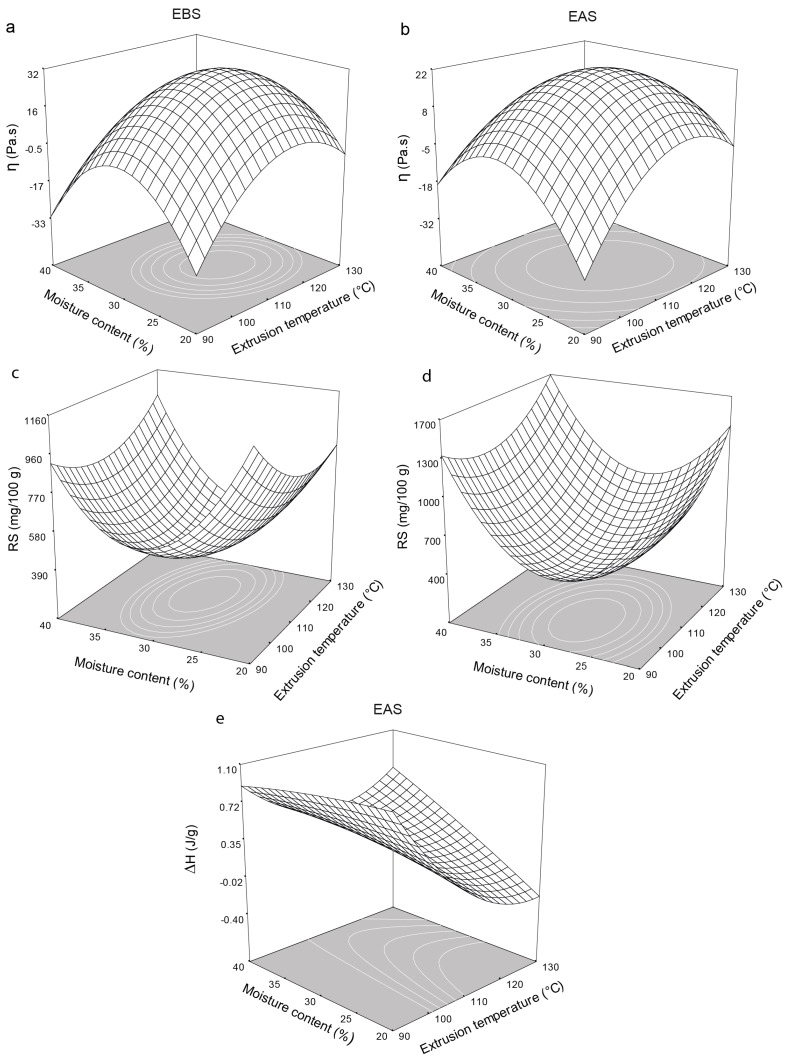
Effect of extrusion temperature and moisture content on apparent viscosity (η), resistant starch (RS) and enthalpy (∆H) at screw speed of 280 rpm on extruded flours before the storage (EBS) and extruded flours after the storage at 4 °C for 120 h (EAS). (**a**)—η of EBS, (**b**)—η of EAS, (**c**)—RS of EBS, (**d**)—RS of EAS, (**e**)—∆H of EAS.

The storage modulus, G’, is a measure of the amount of energy stored in materials and recovered during cycling, which is indicative of the solid or elastic characteristic of the materials. Another parameter that indicates the physical behavior of a system is the loss modulus (G″), which shows the liquid or viscous behavior [42,43]. The extrusion temperature, moisture content, and screw speed significantly (*p* < 0.05) affected both G′ and G″ for the EBS and EAS. Figure 2a,b shows the effects of the extrusion temperature and moisture content on the storage modulus (G′) of the EBS and EAS, respectively. G′ for both the EBS and EAS showed similar trends with increasing extrusion temperature and moisture content. G′ increased to a maximum of 225.87 Pa for the EBS containing 29.34% moisture and extruded at 110.46 °C and 277.98 rpm (Figure 2a) and increased to a maximum of 57.83 Pa for the EAS containing 27.6% moisture and extruded at 117.7 °C and 279.26 rpm (Figure 2b). G″ for both the EBS and EAS also showed similar trends with increasing extrusion temperature and moisture content. G″ increased to a maximum of 45.53 Pa for the EBS containing 28.9% moisture and extruded at 110.92 °C and 277.15 rpm, which is similar to the values for the EAS, indicating that storage had minimal effects on the viscous behavior of the extrudates. The relationship between the two moduli was determined through tan δ = G″/G′, which is a useful indicator of the relative contributions of the viscous (G″) and elastic (G′) components to the viscoelastic properties of a material. The extrusion temperature and moisture content both significantly (*p* < 0.05) affected tan δ for both the EBS and EAS, as shown in Table 5. G′ and G″ both increased with increasing extrusion temperature (Figure 2f). The tan δ reached a saddle point of 0.20 for the EBS containing 28.94% moisture and extruded at 116.62 °C and 309.52 rpm and a minimum of 0.06 for the EAS containing 33.62% moisture and extruded at 116.79 °C and 286.57 rpm (Figure 2e). The low values of tan δ indicate that the material was more elastic than viscous [43]. The magnitudes of the elasticity and viscosity depend not only on the density, rigidity, and spatial distribution of the granules and on the effective contacts between the granules [43] but also on the physical transformations and on the extent of starch molecule rupture generated during extrusion [42]. These low values of tan δ also are related with RS formation, WAI, WSI and η, since to under the same predicted conditions by the model for low values of tan δ lower RS, apparent viscosity and WSI values (Figure 3b,d and Figure 1d, respectively), and high values of WAI (Figure 1b) were observed. In addition, these behaviors were related with the prediction of a maximum value for G′ (Figure 2b).

The extrusion temperature and moisture content also significantly (*p* < 0.05) affected the apparent viscosity (η) of the EBS and EAS. The apparent viscosity increased to a maximum of 31.36 Pa·s for the EBS containing 28.46% moisture and extruded at 113.52 °C and 271.8 rpm (Figure 3a) and to a maximum of 21.59 Pa·s for the EAS containing 29.16% moisture and extruded at 112.77 °C and 278.58 rpm (Figure 3b) with increasing extrusion temperature and moisture content. Successive increases or decreases in the extrusion temperature and moisture content reduced η due to starch degradation during processing [25,38] or to low starch gelatinization [24,37], respectively. This behavior also was found in WAI, having a high relation with the rheological parameters (G’, G” and η) obtained. Since at increases or decreases in the extrusion temperature and moisture content the WAI decreased due to low viscosity obtained under these conditions.

### 2.6. Resistant Starch (RS) Content

The moisture content, extrusion temperature, and screw speed all significantly affected the amount of RS generated in the EBS and EAS (Table 4) through the quadratic effect. The fitted data model predicted that a significant amount of RS would form in the EBS, which explains the estimated 85% of the variability in the data. The adjusted second-order model for the EAS, however, did not show any significant fit, which explains 73% of the variability in the data. Figure 3c shows the effects of the extrusion temperature and moisture content on the formation of RS in the EBS, reaching a minimum of 0.380 g/100 g for the EBS containing 30.3% moisture and extruded at 112.8 °C and 276.8 rpm.

Successive increases or decreases in the extrusion temperature and the moisture content increased the amount of RS (0.960–1.100 g/100 g) formed in the EBS. The formation of RS in the EAS showed a similar trend and was significantly affected by the screw speed. The EAS containing 29.4% moisture and extruded at 101.6 °C and 286.4 rpm contained a minimum of 0.400 g/100 g of RS (Figure 3d) because the shear stress during extrusion provoked the amylose molecules to break down thereby producing less-polymerized starch molecules, which could not be incorporated into the crystal structure thereby decreasing the amount of RS formed [20,35]. Moreover, other factors such as residence time have been associated with low levels of RS formation. The starch structure is completely damaged during long residence times because the solubilization of amylose molecules and the swelling of the starch granules causes the starch grains to lose their crystallinity [23,32] thereby leading to low RS formation. At higher extrusion temperatures and moisture contents, the model predicts that more RS (1.300–1.700 g/100 g) (Figure 3d) will form. The formation of RS in the EAS (Figure 3d) is attributed either to better nucleation and elongation of amylose and amylopectin chains, which facilitate recrystallization, or to retrogradation [20,32,44,45], where starch molecules may reassociate and form tightly packed structures stabilized by hydrogen bonding [16]. For this reason the water absorption and viscosity decreased, having relation with the low WAI and rheological parameters values (G’, G” and η) obtained. The best yield of RS obtained in the EAS was 1.134 g/100 g, corresponding to an increase of 68% compared to native starch. This finding show that the extrusion process combined with storage at low temperature is a viable alternative for RS production, through an environment friendly process. This amount of RS was similar to those previously reported by Gonzalez-Soto et al. and Hagenimana et al. [23,25] who obtained RS yields of 0.70–1.30 and 0.90–3.10 g/100 g from cornstarch and rice starch, respectively, but is higher than those previously reported by Faraj et al. [20] who obtained RS yields of 0.017–0.050 g/100 g from extruded barley flour. As can be seen in Table 2 the increment of RS was higher in the most treatments stored at low temperature (EBS) compared to EAS treatments, generating an average increase of 26%.

### 2.7. Thermal Properties

**Figure 4:**
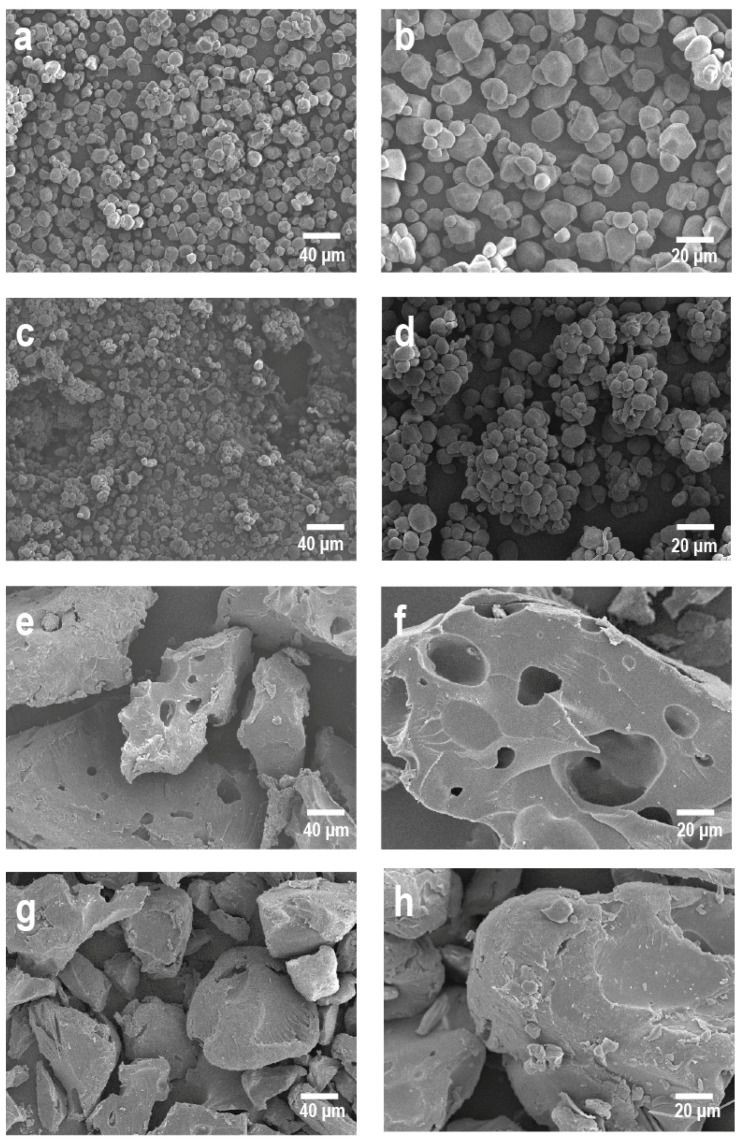
Micrographics SEM of cornstarch (**a**—200×, **b**—750×), standard resistant starch 52% (**c**—200×, **d**—750×) and extruded starch (**e**/**g**—200×, **f**/**h**—750×).

The gelatinization enthalpy is a measure of the crystallinity of the amylopectin, indicating the quality and quantity of the starch crystals [46], and of the amount of energy required for disrupting the H-bonding within junction zones [47]. Two endothermic transitions were found for the native cornstarch: the first corresponding to starch gelatinization and the second to RS formation. Tg and ∆H for the first endothermic transition for the native cornstarch were 69.89 °C and 9.33 J/g, respectively. Similar results have previously been reported [39,42,48,49]. For the second endothermic transition, Tg and ∆H were 102 °C and 0.06 J/g, respectively. The native starch showed high enthalpies because it had not been extruded. The extrusion temperature and the moisture content and the screw speed and extrusion temperature significantly affected the gelatinization enthalpy of the EAS through linear and quadratic effects, respectively (Table 5). The proposed model adequately estimated the data variability. Figure 3e shows the ∆H of the EAS containing different amounts of moisture and extruded at different temperatures. At low temperatures and all the moisture contents, the gelatinization enthalpies increased (0.9 J/g) while at the high temperature, ∆H decreased to 0.38 J/g. The decrease in enthalpy was due either to starch dextrinization or to macromolecules fractionating under the low moisture levels and high temperatures during shear processing [5,42,50] and has previously been associated with the melting of an amorphous amylose-lipid complex [26], as shown in a microscopic analysis (Figure 4h).

### 2.8. Scanning Electron Microscopy (SEM)

Figure 4a,b shows ~15-micron-diameter native cornstarch granules with smooth, defect-free, round or elliptical surfaces, which do not show any signs of damage [1,26,42,51]. Figure 4c,d shows compactly agglomerated 20–25-micron-diameter starch granules containing 52% RS. Figure 4e,f shows how in the extruded starch, the granular structure of native starch is destroyed during the ECP as a result of high temperatures, stress, and pressure, producing gelatinization and starch dextrinization [20,32,52,53], which are the main mechanisms of starch fragmentation during the ECP [5,42,50]. Amorphous, porous structures were formed in the EAS because the pressure difference between the extrudate leaving the extruder and the starch still inside the extruder allowed water molecules to evaporate from the extrudate [49], as shown in Figure 4f for 20-micron-diameter EAS granules.

The amylose leached out of the starch granules during extrusion cooking and bound to the surface of the extruded product, as shown in Figure 4h [54]. The starch granules became more irregular and appeared less granular as a consequence of gelatinization, which had provoked coupled starch granules to form a sponge-like structure within the inner region of the retrograded starch [52]. In contrast, the RS showed increased crystallinity and formed a larger, more compact laminiplantation structure [17].

### 2.9. Identification of Optimum Conditions

**Figure 5:**
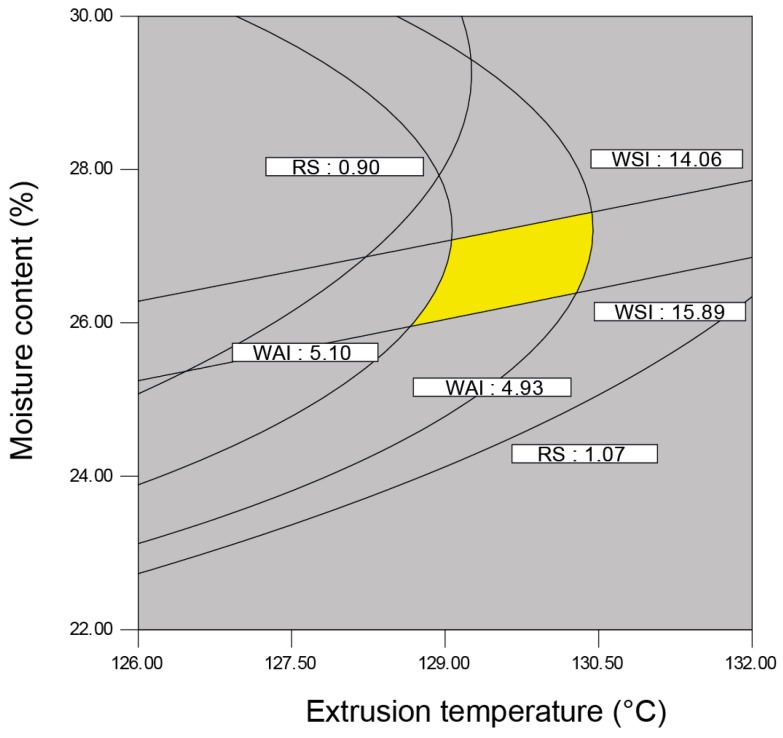
Optimized region obtained of contour plots of the physicochemical characteristics of the flours obtained by extrusion (WAI = water absorption index, WSI = water solubility index, RS = resistant starch).

Canonical analysis [39] was applied to locate the stationary points for the RS, WAI, and WSI responses. An optimization graph of the multianswers was applied [55,56], and the values were grouped and selected for the general optimization of this process. The criterion for the graphic optimization was to find the conditions under which the RS contents were the highest while maintaining the highest WAI and the lowest WSI. The region satisfying this criterion served as a basis for determining the optimum conditions for the process. The overlaid contour plots of the individual RS, WSI, and WAI plots resulted in an optimal region (Figure 5). The predicted optimum conditions were as follows: extrusion temperature (129–131 °C), moisture content (25%–27%), and screw speed (280 rpm), which generated 0.920–0.990 g/100 g for RS content, 3.81–4.98 for the WAI, 21.06 and 19.4–22.23 for the WSI; η = 1.77–3.81 Pa·s, G′ = 42.33–48.46 Pa, and G″ = 7.27–13.83 Pa for the rheological properties; and 0.08–0.13 J/g for the gelatinization enthalpy.

The identified optimum conditions were verified by performing an independent experiment with the EAS. Table 6 shows the experimentally obtained values and the values estimated by the model for the optimization process. The amounts of error between the experimentally obtained values and the values estimated by the model were 9.09%, 10.33%, and 3.41% for the RS content, the WAI, and the WSI, respectively.

## 3. Materials and Methods

### 3.1. Raw Material

The cornstarch (GPC^TM^, Muscatine, IA, USA) used for this study was analyzed for moisture, protein, fat, crude fiber, and ash contents according to methods 934.06, 920.52, 920.152, 945.16, 940.26, and 962.09, respectively [57]. The carbohydrate content was obtained by difference. Additionally, the RS content, apparent viscosity (η), storage modulus (G′), loss modulus (G″), the ratio of loss modulus/storage modulus (tan δ) gelatinization enthalpy (∆H), gelatinization temperature (Tg), water absorption index (WAI), and water solubility index (WSI) were determined.

### 3.2. Chemicals and Reagents

Maleic acid and sodium azide were purchased from Sigma-Aldrich (St. Louis, MO, USA). A RS assay kit including a standard resistant starch with 52% of RS was obtained from Megazyme (Wicklow, Ireland). The other analytical-grade solvents and reagents used for the extractions were obtained from J.T. Baker (Mexico City, Mexico).

### 3.3. Extrusion Cooking

Starch whose initial moisture content was 10.9% was fed into an extruder at 15.97 kg/h d.b. and was adjusted to 20%, 25%, 30%, 35%, and 40% moisture contents and extruded through a 600-mm-long twin-screw co-rotating pilot scale (BTSK-20/40, Bühler AG, Uzwil, Switzerland), where the length to diameter ratio (L/D) = 20. The die diameter was 4 mm, and the screw configuration was specifically selected to create high levels of shear, the first section contained only conveying elements, with the next containing both conveying and kneading elements. Finally, the high-shear section contained conveying, reverse conveying, and kneading elements [58], according to the method described by Ruiz-Gutiérrez et al. [59]. The starch was extruded at 90, 100, 110, 120, and 130 °C, and the extrusion temperature was controlled using a TT-137N water heater (Tool-temp, Sulgen, Switzerland) at the end stage of the extruding chamber. Further, the 180, 240, 280, 320, and 360 rpm screw speeds were evaluated according to the experimental design listed in Table 2. For increase the levels of RS, the extrudates were stored at 4 °C for 120 h in plastic bags and later air-dried at 50 °C for 8 h in a convection oven.

### 3.4. Extruded Cornstarch Flours

The extruded cornstarch was milled (Hammer mill, Pulvex model 200, Mexico City, Mexico), sieved with a 0.8-mm sieve, and re-sieved with an 80-mesh (177-micron) sieve to obtain uniform EBS and EAS particles, which were stored in plastic bags until the analysis.

### 3.5. Physical and Chemical Analyses of Extruded Products

#### 3.5.1. Water Absorption Index (WAI) and Water Solubility Index (WSI)

The WAI and WSI were determined in triplicate according to the method described by Anderson et al. [60]. Briefly, 2.5-g samples were weighed, placed in plastic tubes, mixed with 30 mL of distilled water at 30 °C, and shaken on a vortex mixer for 30 min. The resulting slurries were centrifuged at 3000× *g* for 15 min (Thermo IEC model CL3-R, Waltham, MA, USA). The supernatant was decanted and evaporated at 100 °C until dry. The residue from the supernatant and the sediment remaining in the tubes after decanting the supernatant were weighed. The ratio of the sediment-forming solids to the soluble solids was measured as g water/g sample.

#### 3.5.2. Thermal Analysis

Thermal analysis by differential scanning calorimetry (DSC) was used to determine the gelatinization temperature and the enthalpy of starch gelatinization of the EAS, following the method used by Hasjim and Jane [13]. Two mg of EAS was placed in a pan with 20 µL of distilled water. The pan was sealed hermetically; an empty pan was used as a reference. A calorimeter from TA Instruments (Q-200, Crawley, UK), with a program of 30 to 150 °C and a temperature ramp of 10 °C/min was used. The obtained thermograms were analyzed with universal analysis software (TA Instruments, Crawley, UK) to determine the gelatinization temperature (Tg) and gelatinization enthalpy (∆H). Each measurement was performed twice.

#### 3.5.3. Resistant Starch (RS) Content

The RS content was determined using an enzymatic kit (Megazyme, Wicklow, Ireland) and the enzymatic method used by Goñi et al. [61], which is based on the AOAC method 2002.02 [57].

#### 3.5.4. Rheological Properties

The rheological properties (G′, G″, and tan δ) were determined using a rheometer (TA Instruments AR 2000EX, Crawley, UK). Starch slurry containing 10% solids was used. According to the moisture content of each sample, a volume of water was added gradually, and the mixture was stirred in a beaker until a semifluid mass was obtained. The instrument was equipped with stainless steel parallel-plate geometry (40 mm diameter). The sample was covered completely with the plate, and excess sample was eliminated. All measurements were carried out at 25 °C and a 1000-micron gap. To determine the linear viscoelastic region, strain amplitude sweeps were run from 0.1% to 5% at a frequency value of 1 Hz. The analyzed samples presented similar response with 2% strain. The flow sweep was performed at a shear rate (γ) of 0.1 to 15 1/s thereby relating shear stress (σ) and shear rate (γ) to calculate the apparent viscosity (η) using Herschel Bulkley model [62] at shear rate value of 1 1/s. Oscillatory tests were performed at 25 °C over the frequency range of 0.1–100 Hz, with strain amplitude constant of 2% for all samples. The comparison between treatments was carried out at 2.51 Hz where ∆tan presented a constant behavior [63,64]. Using the above approach, it was possible to determine the dynamic storage shear modulus (G′), the loss shear modulus (G″), and tan δ (i.e., G″/G′).

#### 3.5.5. Scanning Electron Microscopy

The EBS and EAS samples, whose particle sizes <0.15 mm and whose moisture contents were 1%, were stuck to stubs and coated with a gold layer under high vacuum in a Denton vacuum evaporator (Desk II) set to 7.031 × 10^−2^ kg/cm^2^. The samples were examined using a scanning electron microscope (JSM-5800LV, JEOL, Akishima, Japan) equipped with a secondary electron detector operated at an acceleration rate of 10 kV.

### 3.6. Experimental Design and Statistical Analysis

A second-order, three-variable, five-level, central-composite design was used, resulting 20 treatments, which axial and central points were done by duplicate. The results were analyzed using response surface methodology [65]. The feed moisture content, extrusion temperature, and screw speed were independent variables. The fitted second-order polynomial is given by:

(1) $$Y_{i}=b_{0}+\sum_{j=1}^{3}b_{j}X_{j}+\sum_{j=1}^{3}b_{jj}X_{j}^{2}+\sum_{j=1}^{2}\sum_{k=j+1}^{3}b_{jk}X_{j}X_{k},$$

where ***Y_i_*** is the predicted response, ***X_i_*** and ***X_j_*** are the input variables, ***b_0_***, ***b_i_***, ***b_ii_***, ***b_ij_*** are regression coefficients of the intercept, linear effects, squared effects, and interactions respectively. The variable combination is shown in Table 7. Analysis of variance, regression, and canonical analysis for the nature of the response variable were performed on Design Expert software v. 6.01 (Stat-Ease Inc. 2001, Minneapolis, MN, USA) and Minitab Release 14.1 software (Minitab Inc., State College, PA, USA). Significant differences were defined as (*p* < 0.05).

## 4. Conclusions

Extrusion is an alternative method of producing RS. The starch moisture content and the extrusion temperature significantly changed the physical properties of the native cornstarch by increasing the WAI and WSI and decreasing the viscosity and enthalpy because of the ECP-induced damage in the starch. The EAS previously extruded at 120 °C and a screw speed of 240 rpm from starch containing 35% moisture contained the most RS (1.134 g/100 g). Despite not generating high RS content, the ECP produced 68% more RS than that which naturally occurs in native cornstarch with the added advantages of the ECP being a high-productivity, faster, energy-efficient, continuous process.

## Figures and Tables

**Table 1:** Physicochemical characteristics of native cornstarch.

Parameter	Content
Moisture content (%)	10.9 ± 0.29
Proteins (%)	0.10 ± 0.06
Fat (%)	0.05 ± 0.03
Ash (%)	0.03 ± 0.01
Resistant starch (g/100 g)	0.67 ± 0.022
Apparent viscosity (Pa∙s)	0.89 ± 0.015
Storage modulus (Pa)	0.50 ± 0.02
Loss modulus (Pa)	0.47 ± 0.08
The ratio of loss modulus/storage modulus	0.87 ± 0.15
Enthalpy (J/g)	9.91 ± 0.80
Gelatinization temperature (°C)	72.40 ± 0.07
Water absorption index	1.80 ± 0.01
Water solubility index	0.13 ± 0.05

Values are the average of triplicate measurements ± standard deviation.

**Table 2:** Physicochemical and thermal properties of extruded cornstarch at different process conditions.

T	A	B	C	Physicochemical Properties						Thermal Properties	
				EBS			EAS			EAS	
				WAI	WSI	RS	WAI	WSI	RS	∆H	Tg
1	100	25	240	5.54 ± 0.69	13.78 ± 1.68	0.604 ± 0.017	5.75 ± 0.31	10.96 ± 1.46	0.632 ± 0.064	0.59 ± 0.008	101.65 ± 0.89
2	120	25	240	4.90 ± 0.26	16.47 ± 2.10	0.540 ± 0.014	5.86 ± 0.08	14.23 ± 0.58	0.930 ± 0.040	0.06 ± 0.009	92.89 ± 1.31
3	100	35	240	4.41 ± 0.04	5.48 ± 0.23	0.638 ± 0.051	3.08 ± 0.33	4.78 ± 0.19	0.823 ± 0.013	0.43 ± 0.100	100.71 ± 1.55
4	120	35	240	4.90 ± 0.13	4.26 ± 0.67	0.559 ± 0.020	2.99 ± 0.12	4.11 ± 0.14	1.134 ± 0.028	0.46 ± 0.050	101.27 ± 0.23
5	100	25	320	4.74 ± 0.60	28.04 ± 4.52	0.681 ± 0.015	5.10 ± 0.16	13.71 ± 1.12	0.694 ± 0.010	0.69 ± 0.040	101.25 ± 0.28
6	120	25	320	4.42 ± 0.04	28.17 ± 1.88	0.572 ± 0.041	4.69 ± 0.16	20.11 ± 3.13	0.936 ± 0.024	0.01 ± 0.009	95.12 ± 0.74
7	100	35	320	4.77 ± 0.07	4.77 ± 0.20	0.625 ± 0.013	2.90 ± 0.20	2.90 ± 0.04	0.975 ± 0.017	0.79 ± 0.030	101.01 ± 0.95
8	120	35	320	5.40 ± 0.10	7.40 ± 0.65	0.576 ± 0.045	2.79 ± 0.33	5.14 ± 0.39	0.845 ± 0.036	0.08 ± 0.040	94.61 ± 0.36
9	90	30	280	6.02 ± 0.09	9.14 ± 0.40	0.488 ± 0.032	4.07 ± 0.01	6.20 ± 1.10	0.465 ± 0.024	0.92 ± 0.200	100.81 ± 0.79
10	130	30	280	4.56 ± 0.01	10.51 ± 0.21	0.502 ± 0.056	5.50 ± 0.02	10.47 ± 0.64	0.651 ± 0.019	0.38 ± 0.010	100.84 ± 0.91
11	110	20	280	4.37 ± 0.20	28.65 ± 1.58	0.791 ± 0.017	3.71 ± 0.20	17.54 ± 1.75	0.830 ± 0.015	0.01 ± 0.008	101.66 ± 0.33
12	110	40	280	4.12 ± 0.03	3.39 ± 0.70	0.679 ± 0.026	2.78 ± 0.21	4.67 ± 0.14	1.049 ± 0.020	0.53 ± 0.001	100.67 ± 1.91
13	110	30	200	6.45 ± 0.06	8.14 ± 0.78	0.503 ± 0.021	4.72 ± 0.06	3.96 ± 0.10	0.563 ± 0.025	0.39 ± 0.060	100.36 ± 1.23
14	110	30	360	5.77 ± 0.50	10.47 ± 0.21	0.492 ± 0.011	4.95 ± 0.06	6.19 ± 0.19	0.564 ± 0.033	0.38 ± 0.030	98.25 ± 1.05
15	110	30	280	6.76 ± 0.63	8.18 ± 0.14	0.323 ± 0.014	5.06 ± 0.23	4.46 ± 0.32	0.422 ± 0.022	0.21 ± 0.010	101.63 ± 0.14
16	110	30	280	6.21 ± 0.06	5.75 ± 1.76	0.405 ± 0.029	5.89 ± 0.27	4.85 ± 0.41	0.407 ± 0.035	0.26 ± 0.003	100.29 ± 0.60
17	110	30	280	6.52 ± 0.58	6.06 ± 0.90	0.388 ± 0.020	6.17 ± 0.28	6.14 ± 0.17	0.452 ± 0.025	0.24 ± 0.020	98.22 ± 0.28
18	110	30	280	6.29 ± 0.15	6.02 ± 1.12	0.419 ± 0.033	6.53 ± 0.16	8.73 ± 0.42	0.416 ± 0.010	0.39 ± 0.060	99.15 ± 0.49
19	110	30	280	6.12 ± 0.20	7.14 ± 0.13	0.394 ± 0.021	5.58 ± 0.31	5.24 ± 0.70	0.325 ± 0.025	0.35 ± 0.050	99.82 ± 0.53
20	110	30	280	6.93 ± 0.39	7.42 ± 0.26	0.323 ± 0.012	6.81 ± 0.05	8.11 ± 1.16	0.352 ± 0.019	0.32 ± 0.002	98.87 ± 0.27

Values are the average of triplicate measurements ± standard deviation. T = Treatment; A = Extrusion temperature (°C); B = Moisture content (%); C = Screw speed (rpm); EBS = Extruded flours before the storage; EAS = Extruded flours after the storage at 4 °C for 120 h; WAI = Water absorption index; WSI = Water solubility index; RS = Resistant starch (g/100 g); ∆H = Enthalpy of gelatinization (J/g); Tg = Gelatinization temperature (°C).

**Table 3:** Rheological properties of extruded cornstarch at different process conditions.

T	A	B	C	EBS				EAS			
				G′	G″	tan δ	η	G′	G″	tan δ	η
1	100	25	240	107.67 ± 1.07	24.93 ± 0.23	0.23 ± 0.001	16.24 ± 0.42	0.81 ± 0.19	0.66 ± 0.09	0.73 ± 0.064	0.11 ± 0.01
2	120	25	240	59.09 ± 1.48	18.97 ± 1.42	0.32 ± 0.030	27.05 ± 1.23	49.16 ± 9.27	17.85 ± 3.17	0.49 ± 0.019	11.51 ± 0.22
3	100	35	240	0.53 ± 0.34	0.37 ± 0.08	0.72 ± 0.179	0.08 ± 0.03	0.64 ± 0.23	0.53 ± 0.18	0.69 ± 0.152	0.073 ± 0.01
4	120	35	240	1.57 ± 0.58	1.69 ± 0.31	0.90 ± 0.610	0.80 ± 0.03	0.85 ± 0.26	0.43 ± 0.21	0.50 ± 0.104	0.17 ± 0.15
5	100	25	320	55.00 ± 21.0	17.34 ± 4.46	0.32 ± 0.042	6.36 ± 0.31	1.37 ± 0.21	1.29 ± 0.38	0.93 ± 0.148	0.20 ± 0.40
6	120	25	320	27.76 ± 3.01	13.76 ± 2.46	0.46 ± 0.020	10.64 ± 0.11	30.06 ± 3.93	14.97 ± 0.43	0.77 ± 0.087	6.26 ± 0.38
7	100	35	320	1.00 ± 0.42	1.21 ± 0.52	0.94 ± 0.138	1.03 ± 0.14	0.57 ± 0.08	0.35 ± 0.21	0.65 ± 0.157	0.09 ± 0.01
8	120	35	320	26.07 ± 6.50	11.20 ± 1.85	0.43 ± 0.066	2.46 ± 0.09	0.43 ± 0.11	0.22 ± 0.58	0.32 ± 0.013	0.22 ± 0.09
9	90	30	280	19.35 ± 2.70	8.89 ± 1.92	0.50 ± 0.006	1.75 ± 0.15	1.06 ± 0.02	1.57 ± 0.06	1.48 ± 0.079	0.30 ± 0.02
10	130	30	280	100.41 ± 3.80	26.96 ± 1.17	0.26 ± 0.010	19.79 ± 2.36	42.81 ± 1.66	13.95 ± 0.33	0.26 ± 0.001	9.46 ± 0.02
11	110	20	280	21.80 ± 1.76	11.01 ± 0.93	0.50 ± 0.014	1.71 ± 0.18	8.23 ± 0.84	6.36 ± 0.19	0.77 ± 0.066	1.77 ± 0.06
12	110	40	280	1.16 ± 0.36	0.89 ± 0.30	0.82 ± 0.013	0.44 ± 0.02	0.46 ± 0.15	0.52 ± 0.05	0.36 ± 0.005	0.31 ± 0.06
13	110	30	200	99.46 ± 5.60	29.10 ± 2.05	0.29 ± 0.009	9.57 ± 0.73	0.43 ± 0.06	0.64 ± 0.09	1.49 ± 0.082	0.31 ± 0.02
14	110	30	360	109.58 ± 8.62	26.96 ± 2.53	0.25 ± 0.013	15.75 ± 0.21	30.75 ± 2.15	13.09 ± 1.12	1.17 ± 0.251	1.96 ± 0.16
15	110	30	280	291.10 ± 7.63	56.41 ± 1.70	0.21 ± 0.004	32.84 ± 1.85	45.09 ± 3.28	55.85 ± 3.10	0.20 ± 0.001	22.11 ± 0.40
16	110	30	280	211.43 ± 24.1	40.72 ± 2.67	0.21 ± 0.005	29.52 ± 2.77	44.40 ± 4.15	37.40 ± 1.73	0.20 ± 0.010	20.43 ± 0.42
17	110	30	280	234.00 ± 23.5	48.83 ± 0.74	0.19 ± 0.009	25.11 ± 0.12	60.56 ± 5.32	35.63 ± 2.31	0.17 ± 0.003	23.43 ± 0.05
18	110	30	280	289.00 ± 2.82	39.34 ± 2.02	0.18 ± 0.011	33.87 ± 0.67	60.46 ± 3.23	52.50 ± 0.21	0.17 ± 0.003	21.06 ± 0.70
19	110	30	280	316.40 ± 22.5	53.31 ± 0.40	0.19 ± 0.009	37.47 ± 2.04	57.23 ± 2.70	31.77 ± 0.25	0.18 ± 0.007	23.32 ± 0.33
20	110	30	280	263.28 ± 27.9	51.30 ± 5.18	0.22 ± 0.003	28.35 ± 1.18	60.92 ± 2.26	36.11 ± 0.84	0.18 ± 0.006	22.10 ± 0.67

Values are the average of triplicate measurements ± standard deviation. T = Treatment; A = Extrusion temperature (°C); B = Moisture content (%); C = Screw speed (rpm); EBS = Extruded flours before the storage; EAS = Extruded flours after the storage at 4 °C for 120 h; G′ = Storage modulus (Pa); G″ = Loss modulus (Pa); tan δ = The ratio of loss modulus/storage modulus; η = Apparent viscosity (Pa∙s).

**Table 4:** Analysis of variance of physicochemical properties of cornstarch extruded at different condition process.

Source	DF	Mean Squares															
		Physicochemical Properties						Rheological Properties								Thermal Properties	
		EBS			EAS			EBS				EAS				EAS	
		WAI	WSI	RS	WAI	WSI	RS	G′	G″	tan δ	η	G′	G″	tan δ	η	∆H	Tg
Model	9	1.41 *	127.30 *	0.030 *	2.74 *	44.84 *	0.097 *	22,390.78 *	603.56 *	0.10 *	308.26 *	1151.80 *	620.72 *	0.35 *	188.81 *	0.12 *	6.43
A	1	0.48	3.04	0.004	0.35	24.46	0.075	789.77	82.14	0.021	177.78	1612.21	191.83	0.70 *	81.07	0.55 *	26.72
B	1	0.024	827.28 *	0.003	8.27	287.51 *	0.068	4278.16	407.39	0.33 *	213.5	557.36	126.07	0.16 *	26.15	0.13 *	1.38
C	1	0.2	68.27 *	5 × 10^−4^	0.19	9.36	0.004	94.09	2.85	0.0007	8.86	108.24	30.96	0.009	0.2	1 × 10^−5^	4.78
AB	1	0.54	0.25	2 × 10^−4^	0.001	8.2	0.017	1298.74	54.3	0.38	20.94	740.65	120.85	0.001	37.14	0.038	10.27
AC	1	0.028	0.21	4 × 10^−5^	0.036	4.57	0.03	257.15	15.25	0.51	4.23	50.03	1.58	0.0003	3.54	0.1 *	2.34
BC	1	0.57	69.27 *	0.001	0.26	11.21	0.004	1484.3	66.89	0.029	104.45	40.73	0.43	0.60	3.41	5 × 10^−4^	8.39
A^2^	1	3.15	27.45	0.034 *	3.21 *	11.73	0.1	87,001.11 *	2015.96 *	0.093	797.83 *	2254.50 *	2215.74 *	0.67 *	567.04 *	0.19 *	0.48
B^2^	1	9.52 *	169.13 *	0.24 *	13.86 *	47.63 *	0.64	126,500 *	3526.73 *	0.042 *	1361.98 *	4834.64 *	2754.69 *	0.19 *	819.45 *	0.001	1.26
C^2^	1	0.56 *	21.01	0.036 *	2.99	0.43	0.11 *	57,122.64 *	1005.44 *	0.025	679.09 *	3073.96 *	2323 *	1.93 *	812.36 *	0.012	1.47
Residual	10	0.3	9.86	0.004	0.84	4.77	0.032	38,113.3	123.83	0.26	52.94	182.98	105.05	0.025	13.56	0.014	6.28
Lack of fit	5	0.49	18.78 *	0.007	1.28	6.38	0.062 *	30,418.4	200.15	0.052 *	86.47	302.91	109.78	0.050 *	25.69	0.022 *	11.1 *
Pure error	5	0.1	0.93	0.002	0.41	3.15	0.003	7694.92	47.6	0.0019	19.41	63.05	100.33	0.0002	1.42	0.005	1.45
R^2^		0.81	0.92	0.85	0.75	0.84	0.73	0.84	0.81	0.78	0.84	0.85	0.84	0.92	0.92	0.91	0.47

* Significance (*p* ˂ 0.05). A = Extrusion temperature; B = Moisture content; C = Screw speed; EBS = Extruded flours before the storage; EAS = Extruded flours after the storage at 4 °C for 120 h; WAI = Water absorption index; WSI = Water solubility index; RS = Resistant starch; G′ = Storage modulus; G″ = Loss modulus, tan δ = The ratio of loss modulus/storage modulus; η = Apparent viscosity; ∆H = Enthalpy of gelatinization; Tg = Gelatinization temperature.

**Table 5:** Regression coefficients from second-order model of relationships between response and independent variables for extruded cornstarch.

Coefficient	Physicochemical Properties						Rheological Properties								Thermal Properties	
	EBS			EAS			EBS				EAS				EAS	
	WAI	WSI	RS	WAI	WSI	RS	G′	G″	tan δ	η	G′	G″	tan δ	η	∆H	Tg
b_0_	6.35 *	7.32 *	0.39 *	6.35 *	6.58 *	0.44 *	253.71 *	45.80 *	0.23 *	30.14 *	52.35 *	39.66 *	0.17 *	21.18 *	0.29 *	99.35
b_1_	−0.17	0.44	−0.01	−0.17	1.24	0.06	7.03	2.27	−0.036	3.33	10.04	3.46	−0.21 *	2.25	−0.19 *	−1.29
b_2_	−0.04	−7.19 *	−0.01	−0.03 *	−4.74 *	5 x 10^−4^	−16.35	−5.05	0.14 *	−3.65 *	−5.9	−2.81	−0.10 *	−1.28	0.09 *	0.29
b_3_	−0.11	2.07 *	0.01	−0.11	0.76	−0.04	−2.43	−0.36	−0.006	−0.74	2.6	1.39	−0.024	−0.11	0	−0.55
b_12_	0.26	−0.18	5 × 10^−4^	0.26	−1.01	−0.06	12.74	2.61	−0.069	−1.62	−9.62	−3.89	−0.015	−2.15	0.07	1.13
b_13_	0.06	0.16	−5 × 10^−4^	0.06	0.76	−0.02	5.67	1.38	−0.080	−0.73	−2.5	−0.44	−0.006	−0.66	−0.11 *	−0.54
b_23_	0.27	−2.94 *	−2 × 10^−4^	0.27	−1.18	−0.02	13.62	2.89	−0.060	3.61	2.26	0.23	−0.087	0.65	−0.01	−1.02
b_11_	−0.35	1.04	0.04 *	−0.35	0.68	0.06	−58.82 *	−8.96 *	0.061	−5.63 *	−9.47 *	−9.39 *	0.16 *	−4.75 *	0.09 *	0.14
b_22_	−0.62 *	2.59 *	0.09 *	−0.62 *	1.38	0.16	−70.92 *	−11.84 *	0.13 *	−8.06 *	−13.87 *	−10.47 *	0.086 *	−5.71 *	−0.01	0.22
b_33_	−0.15 *	0.91	0.03 *	−0.15	−0.13	0.06	−47.66 *	−6.32 *	0.032	−5.20 *	−11.06 *	−9.61 *	0.28 *	−5.68 *	0.02	−0.24

* Significance (*p* < 0.05). A = Extrusion temperature; B = Moisture content; C = Screw speed; EBS = Extruded flours before the storage; EAS = Extruded flours after the storage at 4 °C for 120 h; WAI = Water absorption index; WSI = Water solubility index; RS = Resistant starch; G′ = Storage modulus; G″ = Loss modulus, tan δ = The ratio of loss modulus/storage modulus; η = Apparent viscosity; ∆H = Enthalpy of gelatinization; Tg = Gelatinization temperature.

**Table 6:** Experimental and predicted values of the responses variables.

	Experimental	Estimated	% Error
WAI	4.34 ± 0.15	4.84	10.33
WSI	21.06 ± 0.52	20.34	3.41
RS	0.88 ± 0.08	0.96	9.09

Values presented are the average of triplicate of the experiment ± standard deviation.

**Table 7:** Process variables and levels used in the experimental design.

Process Variables	Levels				
	−2	−1	0	+1	+2
Extrusion temperature (°C)	90	100	110	120	130
Moisture content (%)	20	25	30	35	40
Screw speed (rpm)	200	240	280	320	360

## Abbreviations

EBS, Extrusion before storage; EAS, Extrusion after storage for 120 h at 4 °C; ECP, Extrusion-cooking process; G′—loss modulus; G″—-storage modulus; η—apparent viscosity; RS—Resistant starch; WAI—water absorption index; WSI—water solubility index; Tg—gelatinization temperature; ∆H—enthalpy.
